# Learning from Transmasculine Experiences with Health Care: Tangible Inlets for Reducing Health Disparities Through Patient–Provider Relationships

**DOI:** 10.1089/trgh.2019.0054

**Published:** 2020-03-16

**Authors:** Nickolas H. Lambrou, Katherine M. Cochran, Samantha Everhart, Jason D. Flatt, Megan Zuelsdorff, John B. O'Hara, Lance Weinhardt, Susan Flowers Benton, Carey E. Gleason

**Affiliations:** ^1^ Department of Medicine, University of Wisconsin School of Medicine and Public Health, Madison, Wisconsin.; ^2^ Geriatric Research, Education and Clinical Center (GRECC), William S. Middleton Memorial Veterans Hospital, Madison, Wisconsin.; ^3^ University Counseling Services, Norris Health Center, University of Wisconsin-Milwaukee, Milwaukee, Wisconsin.; ^4^ Department of Educational Psychology, School of Education, University of Wisconsin-Milwaukee, Milwaukee, Wisconsin.; ^5^ Institute for Health and Aging, School of Nursing, University of California, San Francisco, San Francisco, California.; ^6^ School of Public Health, University of Nevada, Las Vegas, Nevada.; ^7^ Department of Medicine, Wisconsin Alzheimer's Disease Research Center, University of Wisconsin School of Medicine and Public Health, Madison, Wisconsin.; ^8^ School of Nursing, University of Wisconsin-Madison, Madison, Wisconsin.; ^9^ Institute of Living, Hartford Healthcare, Hartford, Connecticut.; ^10^ Joseph J. Zilber School of Public Health, University of Wisconsin-Milwaukee, Milwaukee, Wisconsin.; ^11^ College of Nursing and Allied Health, Southern University and A&M College, Baton Rouge, Louisiana.

**Keywords:** transgender health, access to care, transmasculine identity, social determinants of health, health disparities, resilience

## Abstract

**Purpose:** We examined health care experiences of transmasculine young adults to clarify factors contributing to mistrust in the health care system and identify tangible and modifiable means to address health disparities through improved patient–provider interactions. Thematic analysis highlights patterns within historical relationships between medical models and transmasculine embodiment, and provides guidance for health care clinicians, researchers, and policy makers to deliver competent services for transgender and gender diverse (TGD) individuals.

**Methods:** The study team used qualitative methodology guided by interpretive phenomenological analysis. Semistructured interviews with 12 participants who self-identified as transmasculine were conducted, transcribed, and coded thematically.

**Results:** Participants were a community sample of 12 young adults 18–35 years of age (*M*=23, standard deviation=3.74), who self-identified as transmasculine. Three participants identified as a racial/ethnic minority. Participants were highly educated, with most completing at least some college. The superordinate thematic domain Perspectives on Health Care emerged, under which three subthemes were nested: (1) an essentialist, binary medical model is inaccurate and oppressive, (2) consequences of medicalizing gender (i.e., gender as a diagnosis), and (3) recommendations to improve health care.

**Conclusions:** Qualitative analysis revealed specific ways in which the relationship between transmasculine individuals and current health care systems are fraught with difficulties, including the impact of stigma, gatekeeping, and inaccuracies, in current diagnostic criteria. Participants shared lived experiences and offered innovative ideas to improve health care delivery, such as challenging socialized biases, increased education, and immersion in TGD communities to advocate for change in research, practice, and policy.

## Introduction

Transgender people are estimated to comprise 1.4 million individuals within the U.S. population.^1^ This is likely an underestimate, considering the stigma associated with reporting transgender and gender diverse (TGD) identities. Moreover, inadequate tracking omits TGD identity designation in research and practice. This stigma and omission may be based in essentialist assumptions, which hold gender identities and expressions as binary, fixed, and entirely derived from biological sex, rather than a diverse or socially constructed phenomenon. That is, the outward appearance of genitalia at birth determines one's sex, gender identity, gender expression, and sexuality.^2–4^ Although the essentialist paradigm can be useful for scientific classification, the application is restrictive when applied to the complexity of sex, gender identity, and expression. Consequently, essentialism disrupts TGD individuals' relationships within the health care system.^3^

Data gathered from the 2010 National Transgender Discrimination Survey (NTDS)^5^ revealed that TGD clients reported negative interactions with providers at staggering rates. Reports included lack of provider knowledge (50%), harassment (28%), refusal of care (19%), and violence in medical settings (2%).^5^ These reports highlighted a substantial gap between best practice and current practice, and suggested that the health care system requires substantial training, transformation, and engagement with the increasing body of empirical literature supporting TGD health to provide competent care for this growing population.

These negative encounters in health care may be underpinned and exacerbated by essentialist assumptions.^6–8^ Furthermore, they exemplify what minority stress theory categorizes as “distal stressors,”^9^ and may have physical ramifications. For example, when encountered, distal stressors can initiate a repeated triggering of autonomic nervous system and hypothalamic-pituitary-adrenal axis responses,^10^ with cumulative effects leading to increased risk for chronic health conditions and accelerated aging (i.e., weathering).^11^ Related to this, research suggests that TGD individuals experience chronic health conditions (e.g., hypertension, diabetes, obesity, stroke, depression, and post-traumatic stress disorder) at higher rates than cisgender peers.^5,12–14^

Effects also play out on a practical level, with 33% of TGD respondents in the NTDS postponing preventive care and 28% avoiding health care altogether for fear of discrimination and mistreatment based on their gender identity.^5^ Another study found 40% of TGD elders feared accessing health care services due to discrimination and internalized stigma (both modifiable factors that intensify chronic stress).^13^ Undoubtedly, health care providers are instrumental to reducing disparities and facilitating favorable health outcomes for TGD people.

Recent diagnostic changes reflect health care's shifting views of TGD identities. Particularly, TGD identities are no longer viewed as a pathological mental illness. Rather, in 2012, the World Professional Association for Transgender Health (WPATH) recommended that clinicians address psychological distress associated with stigma and discrimination when necessary, and ensure access to care based on an informed consent.^15^ In addition, in its 2019 revision to the ICD-11, the World Health Organization (WHO) re-classified “gender identity disorder” as “gender incongruence” and moved it from “Mental and behavioral disorders” to a new chapter entitled “Conditions related to sexual health.”^16,17^ Despite acknowledged imperfections, the WHO posited this revision is a step toward de-pathologizing TGD identities, while allowing access to affirmative care.^17–19^

This project examined health care experiences of transmasculine young adults to clarify factors contributing to minority stress and resilience, and identify ways to improve patient–provider interactions. Researchers extracted data from a larger, overarching qualitative study that highlighted how transmasculine young adults defined the term “transition,” and made meaning around concepts like gender, intersectionality, and the process of transmasculine identity development. Here, themes regarding perspectives on health care are presented. Rather than proceeding with a preconceived hypothesis, we phenomenologically allowed themes to emerge.

## Method

A qualitative approach was used for the overarching study, guided by interpretive phenomenological analysis (IPA).^20^ IPA examines how people make meaning of significant life experiences on individual and group levels, and is based in three philosophical tenets: (1) phenomenology, (2) hermeneutics, and (3) idiography.^20,21^ All research activities were approved by the University of Wisconsin-Milwaukee Institutional Review Board.

Recruitment efforts included personal visits to Midwestern LGBTQ community centers, and outreach to various online social media groups. Overall, 15 people responded to postings, and 12 met inclusion criteria (e.g., 18–35 years of age, self-identifying as transmasculine). Data used in this study were derived from the same 12 participants (Table 1). Reasons for exclusion included not self-identifying as transmasculine, not meeting the age requirement, and interest in participation after data collection concluded. Participants were interviewed in safe settings, including library study rooms, university spaces, and secured online video conferences, such as Zoom.

**Table 1.** Demographics

Participant	Age	Pronouns	Gender identity	Race/ethnicity	Education (highest level)
1	25	He/him/his	Transmasculine	Mixed (e.g., African American, Irish, German, Cherokee, French)	Associates degree
2	23	He/him/his	Transmasculine	White	High school diploma
3	26	He/him/his They/them/theirs	Transmasculine	White	Bachelor's degree
4	19	He/him/his	Transmasculine	Black/African American	Undergraduate college student
5	25	They/them/theirs	Trans Person/Genderqueer/Transmasculine	White	Bachelor's degree
6	26	He/him/his	Transmasculine	Brown/Latinx	Graduate student
7	21	He/him/his	Transmasculine	White	Undergraduate college student
8	18	They/them/theirs He/him/his	Transmasculine	White	Undergraduate college student
9	20	They/them/theirs	Transsexual/Transmasculine	White/Euro American	Undergraduate college student
10	30	They/them/theirs	Transmasculine	White	Doctoral-level student
11	19	He/him/his	Transmasculine	White	Undergraduate college student
12	26	He/him/his	Trans Guy/Transmasculine	White/Irish, Scottish	Bachelor's degree

### Data collection

Data were collected with semistructured, one-on-one interviews between the lead researcher and participants. We selected this approach for flexibility that allowed unexpected, meaningful dialogue to emerge.^22^ Researchers developed an open-ended interview guide to facilitate explorative free-flowing narratives, while remaining focused on topics of inquiry. This format allowed for (1) seamless movement from one topic to another, as unexpected conversational shifts are noteworthy in IPA analysis, and (2) participants as experts, leading researchers to thematic illustrations of “the thing itself.”^20^ Topics broadly explored included identity development, intersectionality, meaning making, and experiences in health care systems.

After obtaining informed consent, the interview process focused on building rapport, while gathering demographic data. Interviews were audio recorded and transcribed verbatim for analysis. After transcription, participants were asked to review their transcript and encouraged to provide feedback. Two participants responded with edits (e.g., elaborations and typo corrections). All approved their transcriptions. Participants received $20 cash remuneration.

### Research team

Two advanced doctoral level counseling psychology students were hand-picked to assist with data analysis, as they had extensive multicultural training, which included issues relevant to TGD identities. In the spirit of reflexivity and transparency, the team discussed intersectionality of their own identities, and how they may shape the analytical lens, which included (but were not limited to) the following: transmasculine, cisgender, female, white, 30–40 years of age, and queer. All team members were experienced qualitative researchers. The lead researcher provided mentorship specific to the IPA approach, emphasizing a safe and open climate, wherein researchers were encouraged to express ideas and challenge one another on potential blind spots. Researchers unanimously agreed that reflexivity, respect, active engagement in difficult dialogues, and bracketing preconceived ideas in analysis were critical to the research process.

### Data analysis

**FIG. 1.**
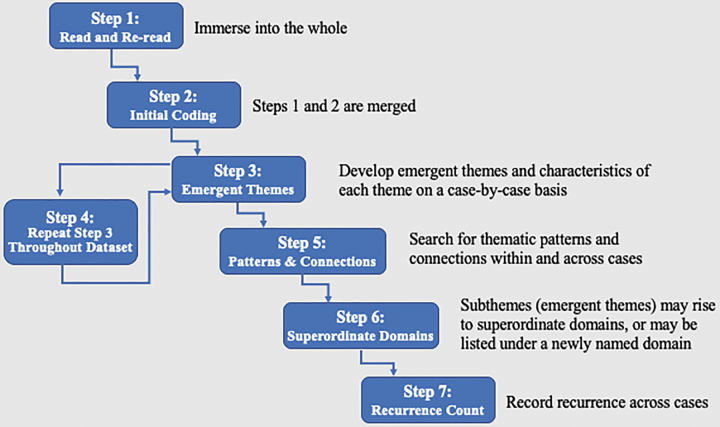
Steps to interpretive phenomenological analysis, adapted from Smith et al.^20^ This figure illustrates the recommended steps researchers followed in the analysis of qualitative data collected for this study.

As per IPA principles, analysis was neither fixed nor rigid, but comprised a common procedures set and commitment to illustrating group-level themes with idiographic, individual experiences.^20^ Because 12 participants was large for an IPA sample size, we developed an analysis plan adapted from Smith et al.,^20^ which adhered to core IPA principles (Fig. 1). The team repeated the steps until saturation was reached.

Our large sample allowed tabulation of cross-case thematic recurrence rates. Measuring recurrence supports the validity of findings, as it quantifies reports of a particular phenomenon across participants.^20^ For this study, designation of superordinate domain was set at a stringent 100%, meaning that subthemes must have appeared in all 12 narratives to meet criterion. The recurrent designation for subthemes was set at a rate of >50%, that is, for a subtheme to become recurrent, it must have appeared in at least 7 of 12 transcripts (Table 2).

**Table 2.** Thematic Recurrence

Superordinate domain: Perspectives on Health Care (100% reporting)													
Emergent theme	Participant												Present in over 50% of sample
	P1	P2	P3	P4	P5	P6	P7	P8	P9	P10	P11	P12	
1. An essentialist, binary medical model is inaccurate and oppressive	X	X	X		X	X	X	X		X	X	X	Yes (83.3%)
2. Consequences of medicalizing gender (i.e., gender as a diagnosis)				X	X	X	X			X	X	X	Yes (58.3%)
3. Recommendations to improve health care	X		X		X	X	X	X	X	X		X	Yes (75.0%)

### Data trustworthiness

**FIG. 2.**
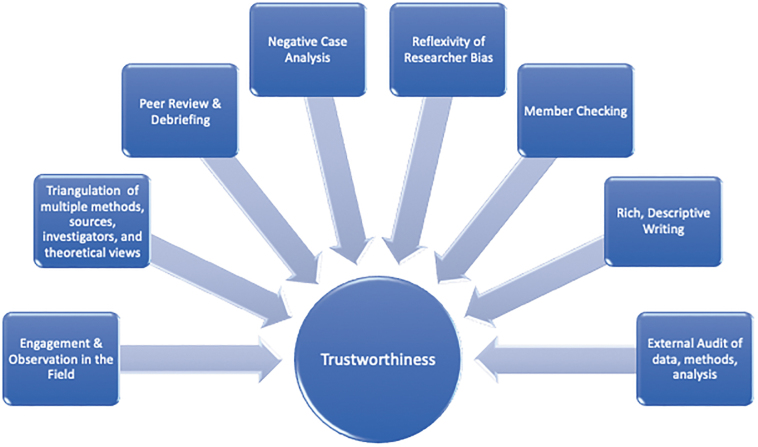
Trustworthiness strategies, adapted from Creswell.^23^ This figure illustrates steps and procedures researchers followed to ensure trustworthiness (i.e., validity) in data collection, analyses, and interpretation.

To broaden trustworthiness (i.e., validity),^23^ individual quotes substantiated themes at the group level. Participant feedback was encouraged to ensure accuracy of data, interpretations, and representativeness of experiences. Researchers continually balanced reflexivity and subjectivity throughout the analytic process. Frequent research meetings allowed expression of multiple viewpoints, expectations, and preexisting notions. Differing narratives on the same topic were examined, with emergent subthemes and superordinate domains calibrated accordingly. Transparency guided the analytic process, and researchers followed steps to demonstrate credibility and applicability^23^ (Fig. 2).

## Results

### Demographic characteristics

A community sample of 12 young adults self-identifying as transmasculine and between 18–35 years of age (*M*=23, standard deviation=3.74) participated in the study (Table 1). Three participants identified as a racial ethnic minority (mixed race/ethnicity, Brown/Latinx, or Black/African American). Participants were highly educated, with most completing at least some college.

### Thematic outcomes

From the full analysis, Perspectives on Health Care arose as a superordinate domain and is the focus of this article. Three subthemes comprise this domain: (1) an essentialist, binary medical model is inaccurate and oppressive, (2) consequences of medicalizing gender (i.e., gender as a diagnosis), and^3^ (3) recommendations to improve health care.

### Subtheme 1: An essentialist, binary medical model is inaccurate and oppressive

Participants described experiences with a health care system that largely reifies an essentialist, binary sex/gender framework, and found case conceptualization through an essentialist framework to be inaccurate and contrary to affirmative health care. For example, participants found binary categorizations of sex and gender erroneously simplistic, leading to the common, fundamental mistake of muddling constructs of sex and gender. These limitations resulted in negative experiences with providers, often leaving participants in the position of educating providers, vulnerable, invalidated, and cautious. In addition, when seeking physical transition-related care (e.g., hormones), participants often received messages that they were “not trans enough” and would be denied care if they did not meet cisgender expectations of men/masculinity. Moreover, participants viewed transition as a lifelong process encompassing many psychosocial facets, rather than a “one and done” treatment. Participants illustrated the impact of systemic oppression (e.g., binary only options on clinical forms), representing a large misalignment between the current health care system and TGD well-being.(Table 3).

**Table 3.** Perspectives on Health Care

Subtheme 1: An essentialist, binary medical model is inaccurate and oppressive		
Subtheme characteristics	Phenomenological interpretations	Participant quotes
Roots of deleterious gatekeeping practices are tied to essentialist, binary assumptions Systemic oppression rooted in essentialist, binary assumptions Pressure to present as “trans enough” by way of essentialist standards to be deemed valid and/or access appropriate health care generates fear, frustration, and anticipated threat Medical intervention assumed to define transitioning process; however, interventions are only one potential facet	Participant 2 demonstrated how the theme of “not trans enough” played out frequently in the context of interactions with health care providers	I'm always kind of concerned that I'll be seen as not trans enough, and they'll be like, “You can't go on testosterone” or something. But that is probably just my brain thinking of worst case scenarios… I don't know. I don't think people have to be hypermasculine. I think everyone should just be themselves. And you shouldn't deny people medical help for that.
	Participant 3 exemplified the idea of “not trans enough,” and binary reification in medical model. Medical interventions are specified as one potential aspect of transition	And if you're a trans man and you never transition medically and stuff… like if you don't even change your name. Maybe you have a gender-neutral name assigned at birth and you never change anything. It's just the same you're still trans.
	Participant 11 reflected on essentialist, binary roots in the medical model and gatekeeping	I know somebody right now who is non-binary and seeking to go on T just for a couple years, but will the doctors even want to treat this person if they express that their end goal is not transitioning? Because the entire process is so focused on, “You want to become cis, don't you?” No, I don't, because that's literally the worst case of anything I could ever be. I don't know, those are some significant things to me, just because everything is deeply rooted in these constructions.
	Participant 7 offered reflections regarding the historical roots of gatekeeping and the invalidation of transmasculine identities	I definitely think that is where [not trans enough] had its roots, because historically if you look at medical transition, trans men were not given access to medical transition because it was only thought of as a trans woman thing. Like, gender dysphoria or being trans was bad. So trans men who said that they were trans and want to transition, it was like, that's not real… that's not a real thing. It was immediately pushed away. People were not allowed to be their full selves if they wanted to be.
Roots of deleterious gatekeeping practices are tied to essentialist, binary assumptions Systemic oppression rooted in essentialist, binary assumptions Pressure to present as “trans enough” by way of essentialist standards to be deemed valid and/or access appropriate health care—generates fear, frustration, and anticipated threat Medical intervention assumed to define transitioning process; however, interventions are only one potential facet	Participant 4 specified medical as one aspect of transition, and demonstrated how “not trans enough” plays out within transgender communities	On Tumblr… I was looking at things, and they were like, ‘Medical transitioning!’ I'm like, “Okay, you got to do that to be this,” because a lot of people wanted it so badly. So, I feel like if they wanted it, it had to be something that was needed for everyone. Until that notion was thrown out the window by co-workers of mine and other trans people I met in person. They were like, “You can still be trans without any medical interventions ever… at all.” I was like, “Oh, cool.”
	Participant 5 pointed to systemic oppression when navigating cisgender frameworks and the necessity of carving out TGD-relevant frameworks	The medical system wasn't set up for trans people. It wasn't set up for queer people. So, trying to etch out a space not made for you is not very easy to do… There's “trans enough” again [laughs]. Am I trans enough for hormones? People pretend they have more dysphoria than they do so they can have access.
	Participant 8 gave practical examples of systemic oppression (e.g., binary only options on medical forms), representing a large misalignment between the current health care system and TGD well-being	But then all the forms that you have to fill out…they did not have anything that was non-binary inclusive. It was all opposite gender, other gender… all that “one or the other.” So, I felt like if I had any inkling of a non-strictly masculine feeling, then [access to appropriate care] would be denied to me.
	Participant 10 specified medical facets as one part of transition, referring to in-group and out-group pressures to conform to binary expectations	I think that was definitely something I thought about more when I first decided to transition, in terms of chest surgery and things like that. But moving along, as I identified more as trans masculine, I realized I was still comfortable with them and I didn't necessarily need to go through a surgery to fully identify with who I am. They weren't keeping me from anything, or inhibiting me from fully identifying with who I am.

TGD, transgender and gender diverse.

### Subtheme 2: Consequences of medicalizing gender (i.e., gender as a diagnosis)

Participants discussed the diagnosis “gender dysphoria,” and resultant stigma around TGD identities. Externalizing stigma was taxing and ongoing. Participants did not view all transmasculine experiences as dysphoric, yet they felt pressure to placate providers by emphasizing dysphoria and/or identifying with cisgender binary standards to access care. Contrary to revised standards of care,^15^ participants reported being “forced” to accept a diagnosis, with practitioners wielding “gatekeeping” power to withhold care. Notably, while the applied effects of gatekeeping emerged in this subtheme, thematic roots of gatekeeping as inextricably tied to essentialist assumptions are captured in the previous subtheme (Table 4).

**Table 4.** Perspectives on Health Care

Subtheme 2: Consequences of medicalizing gender (i.e., gender as a diagnosis)		
Subtheme characteristics	Phenomenological interpretations	Participant quotes
DSM=historical stigma Applied consequences of Gatekeeping vs. Informed Consent models of care Essentialist, binary medical model limits ability to self-actualize, hence, limits well-being Essentialist assumptions giving rise to pathology; both have harmful consequences (e.g., internalized oppression, fear, and apprehension/avoidance to seek health care) Forced to accept stigmatizing diagnosis to access appropriate care Diagnosis may not be accurate; seen as “just another hoop to jump through”	Participant 7 reflected on the implications of changes to DSM diagnoses over time, and oppression rooted in the medicalization of TGD identities and embodiment	The whole system is really complicated. I don't think it should be listed as anything honestly. I do think it is an improvement over gender identity disorder being listed as a mental disorder. Now, where it's gender dysphoria, it has less of a connotation of someone being mentally ill. Rather, they have something that exists and it can be treated if they choose… But I am super against the medicalization of trans bodies, and trans identities, which I still think… the idea of gender dysphoria in the medical setting is really limiting, and it also allows doctors to keep resources from trans people… I am also liking how informed consent is a thing now, which I didn't realize until after I had started my [medico-legal] transition. But the gatekeeping model where [they] have to know if you are mentally ill or not before we give you testosterone… where at some places they're like, ‘You are an adult, here is what it does to your body, feel free to take this medication and keep it monitored.’ So, I think that all medical stuff related to transness is bad [laughs]. It's really limiting.
	Participant 5 illustrated how essentialism and pathology contribute to internalized hatred, and how extra work is needed to reclaim identity and live authentically (self-actualize)—both are core to well-being	We want to popularize the term “Gender Euphoria” cuz you know I feel the gender dysphoria in my body, and the internalized trans phobia that society has taught me. Yeah. It's hard because I have gender identity disorder on my diagnoses because it has to be. I mean, I feel gender dysphoria, but not every trans person does. So, I don't think it should be pathologized, I don't think it's an inaccurate way to describe me (laughs) but it's not an inaccurate way to describe all trans people or even trans people who want hormones or medical transition. And I think there is no reason why queer identity should be in the DSM. We should have learned this lesson already. What makes me mentally ill is *not* that I'm trans! (laughs)
	Participant 6 added a critical aspect of gender as a diagnosis as it pertains to people with multiple marginalized identities	Participant 6: The diagnosis is just pathologizing. It should not even be in the DSM… it is stigmatizing. Informed consent is the way to go. People travel very far distances just to go to a center that has informed consent. They can see therapists that are onsite if they want, but it should not be required to get access to medical transition. Interviewer: Thinking about all the hoops one must jump through to “transition,” or to get legal documents changed. [Interviewer reflected on own bias and privilege when referring to ‘legal documents’]. I can't even imagine what it would be like if English wasn't first language, or if they are undocumented. Participant 6: Yes, and many times it is dangerous for undocumented people to disclose their status to people in health care fields, so they just do not. This is a huge barrier, and very damaging.
DSM=historical stigma Applied consequences of Gatekeeping vs. Informed Consent models of care Essentialist, binary medical model limits ability to self-actualize, hence, limits well-being Essentialist assumptions giving rise to pathology; both have harmful consequences (e.g., internalized oppression, fear, and apprehension/avoidance to seek health care) Forced to accept stigmatizing diagnosis to access appropriate care Diagnosis may not be accurate; seen as “just another hoop to jump through”	Participants 8 offered insight through personal experiences in gatekeeping and incompetent provider care	Oh. My. God. Yes! So, when I talked about hormones with my first “trans expert” therapist, she was… a cis het lady. She had no idea what she was talking about. She had no idea. She just went to some class, and people gave her a certificate that says ‘you're trans friendly’ even though she wasn't… she was like, ‘Oh, your identity has been so fluid lately. You used to identify as non-binary, and I know you had all the same dysphoria and everything, but I would want you to identify as a trans man for six months before I would feel comfortable giving you a letter for hormones.’ That's not okay! I did not schedule another appointment with her after that, but even when I was going in for the psych appointment to get my hormones, luckily the psychiatrist that I had who was evaluating me was really good about it.
	Participant 11 reflected on impact of gatekeeping vs. informed consent models, and the vulnerability of being subjected to inaccurate binary assumptions in DSM diagnostic criteria	I did fib a couple of times. Like, ‘Oh yeah, I feel like the opposite gender. I feel like the body parts of the opposite gender, which is just a really weird and awkward sentence. That could mean so many things. The body parts of the opposite gender… by that, I would assume they mean penises would be more comfortable… I felt like if I answered anything the way they didn't want me to, I wouldn't be able to get what I needed. It takes a long time to get in to even start the process. I hate the process that I had to go through to get on HRT, and throughout that whole process I was being told that I should be thankful it was this easy for me. Like, here at the [college health clinic], they said, ‘You should be glad.’ Whatever. It should be easier. It should be one appointment, like, ‘Do you want this?’ and ‘Yes, give it to me.’
	Participant 10 pointed to how essentialist, binary assumptions are detrimental, and weaken validity of the medical model	I think it can be detrimental to think about it as a mental disorder. I purposely went outside of the state so that I wouldn't have to go through that whole procedure. It just seems wrong to… not medicalize it… but, turn it into a pathology when so much of it is culturally constructed.
	Participant 12 commented on stigma and diagnosis, harmful effects of an essentialist medical model, and stressors associated with accessing appropriate health care	I feel like it's not a disorder, and if it is, then where is my monthly government check? That's what I want to know! I had a friend who lives somewhere horrible, and it is nearly impossible for them to get access to these things. So I told them that you need to tell them… not say that you don't know… or that you feel like “this” one day, and like “that” another day. You have to go in telling people, because it's really hard. I feel like it is important though… and I wish this wasn't a thing, but for ‘transtrenders.’ I couldn't imagine some kid who felt like they were a boy, but really in the long run they are lesbian or non-binary or something like that - to literally physically change themselves just for something they're not too sure about. So, I do think going to therapy is definitely important, but I think that there needs to be more flexibility, and there shouldn't be any Christian therapy.

### Subtheme 3: Recommendations to improve health care

Participants offered practical suggestions for providers. Foremost, participants wanted to trust, and urged providers to listen and challenge personal biases. Providers would garner trust by informing themselves rather than placing that onus on the individual in their care. One suggestion to facilitate self-guided learning included active immersion in TGD communities, and conducting affirmative research in multiple fields of study (e.g., endocrinology, public health, mental health, neuropsychology, and primary care). Interestingly, participants commented that binary constructs also harm cisgender people, restricting variation in identity and expression, and suggested approaching topics of transmasculinity and TGD health with the same respect, curiosity, and fervor as any scientific inquiry. Thus, continual self-reflection, developing TGD-centric frameworks, and challenging socialized biases were described as essential to competent research and practice.

Inclusive clinical forms and environmental cues were important, including diverse décor, competent front-line staff, and witnessing an array of practitioner identities reflecting those of participants. Language was named as a significant catalyst for changing or maintaining the status quo. Participants offered suggestions to expand language, thereby social constructions. For example, abandon binary forced-choice options (e.g., boxes for male/female), add space for pronouns in use, and/or include write-in options for identities in clinical and research demographics (Tables 5 and 6).

**Table 5.** Perspectives on Health Care

Subtheme 3: Recommendations to improve health care		
Subtheme characteristics	Phenomenological interpretations	Participant quotes
Providers not informed: physically and emotionally taxing for participants Challenge systemic oppression (e.g., binary options only on forms, outdated language or practices, need for facilities to be inclusive and welcoming) Societal expectations and provider bias: • toward transitioning (socially and medically) • assessment, presenting concerns, and treatment planning Providers need to listen, validate, and understand TGD people as both vulnerable and resilient. Transmasculine identity is not the core of distress, rather external stressors Transmasculine identity as a source of resilience (e.g., “gender euphoria”) Explore bias and consequences of binary and medicalization Positive patient–provider experiences can increase levels of safety, trust, validation, and access to competent care, thus reducing disparities	Participant 1 described attributes of a good mental health therapist	A good therapist is knowledgeable; wise. If they're young, wise for their years. Experienced. Has connections with all sorts of people so that they have a wider view of who they're reaching. Um, and they're not afraid to ask questions. One that's professional and doesn't breach certain boundaries that… oh oh oh… and at the very beginning they talk with you about boundaries of what you are expecting, of what they can do.
	Participant 5 asserted the critical need for providers to understand TGD people as a vulnerable population, and offered practical recommendations (e.g., explore internal bias, increase positive TGD visibility, and inclusive restrooms) to remedy health disparities	You need to know trans 101, because trans people are a really vulnerable population. There are a lot of young trans mentally ill people who need services where you guys tend to [mess] up. But I'll just be nice about that last part. I think that internal bias is something mental health providers need to deal with because I'm sure there are plenty of mental health providers who are thinking to themselves, like, trans people are inherently unstable or poly relationships are inherently unstable or gay people are promiscuous… providers need to deal with whatever biases they are holding. They need to make their facilities tangibly more like welcoming and comfortable… put up a cheesy poster about how you accept gender diversity or something in your waiting room! Put some *Out* magazines in your waiting room or configure facilities so that people have gender-neutral bathrooms. The way that we culturally made changes for disabled accessibility, I think we need to make those changes for trans accessibility.
	Participant 3 shared negative experiences of being misdiagnosed, exemplified the added burden of having to inform practitioners, and offered applied examples of how to reduce disparities in health care provision.	[Providers] would ask me about myself and about my ex, and when I described [us], I used they/them pronouns. [Providers] were getting so confused. They thought I had dissociative identity disorder…Yeah, if I have to teach you about who I am and how I work then you're probably not the right psychiatrist for me… because you get to talking about trans stuff with them, and you truly feel like you're a little insane or something. You think maybe there is no one else like me, or maybe there are people out there like me, but they also have a crazy brain disease. That's kind of how I felt there. It's always a learning experience even when you have your degree. As medical folks know, there's new diseases being found, there's different types of people out there. There's new genders out there that you didn't realize were there before, but it's been around for quite a while. So just brushing up on that, and not thinking of it as something that's “new age.” Just be more respectful. Medical forms… have an option to put “other.” If someone is trans or not… that's [being informed] actually a big deal. For example, I have a lot of chest pain because I wear very tight sports bras. I never thought of it, and it was my chiropractor who said my back was messed up. She was like, “Do you do any weird stuff with your back?” It's always my middle back, and I said I wear tight sports bras, and she asked why I would do that. I said to make my chest flatter. Then it was this really awkward thing. But, if you're trans and binding all the time, it is really bad for your back. So, having someone in the medical field talk about safe ways to feel better about yourself, that would be really nice… There is such a benefit to knowing someone is trans.
Providers not informed: physically and emotionally taxing for participants Challenge systemic oppression (e.g., binary options only on forms, outdated language or practices, and need for facilities to be inclusive and welcoming) Societal expectations and provider bias: • toward transitioning (socially and medically) • assessment, presenting concerns, and treatment planning Providers need to listen, validate, and understand TGD people as both vulnerable and resilient. Transmasculine identity is not the core of distress, rather external stressors Transmasculine identity as a source of resilience (e.g., “gender euphoria”) Explore bias and consequences of binary and medicalization Positive patient–provider experiences can increase levels of safety, trust, validation, and access to competent care, thus reducing disparities	Participant 5 described what a positive experience looked like for them	My first doctor described it in a way that I really loved. He said that being trans is not an illness, but it's still something that, for some people, can benefit from medical intervention… similar to a pregnancy. You're not sick if you're pregnant, but you still might benefit from accessing medical care. I think that I really benefited from accessing medical care. I don't plan to have any surgeries currently, but I might change my mind. I know when I came out as genderqueer, people were asking, ‘Are you going to start hormones? Are you going to modify your body?’ I was like, ‘Not right now. I'll decide later.’
	Participant 7 demonstrated that, to him, practitioners informing themselves on transmasculine identity would mean he is valuable and cared for	It's not even what I want them to know, it's that I want them to care enough to look for the resources to know. Because there is so much information out there on how to support trans people, and how medical transition works. There are so many articles. I've done a lot of research on how teachers can be inclusive, medical practitioners can be inclusive, and it's like people don't care enough to look up how to do that. They don't care enough to be educated. So I don't even think it's a lack of information, I think it's a lack of giving a shit about being supportive of trans people. Because I've had to go to my doctor and be like, ‘I bind, I know that something is wrong, and I need you to fix it.’ They're like, ‘Well, we've never had a trans person, we don't know what that is.’ I have had to educate them… Google my own symptoms and be like: ‘Other trans men are experiencing this, and this is something I think I have.’ But I'm not a doctor; they are a doctor! Yeah, they need to look to other practitioners who are knowledgeable and give a shit about being inclusive. I forget the statistic but, from the U.S. Trans survey in 2015, a really high percentage of trans individuals don't even seek medical care because they don't want to be disrespected, or they don't want to have to teach their own doctors… that is ridiculous!
	Participant 8 remarked on the bias in mental health care that many presenting concerns are directly related to, and a product of, transmasculine identity	I don't want it to be as big of a deal in therapy … like every single time I go to the therapist, they're like, ‘Okay, so you're transgender…’ and then they take every single issue that I have and frame it around my gender identity, when that's not the case. My gender doesn't make me depressed or anxious. The problem is the way that other people are. There's nothing wrong with me. I don't need therapy for being transgender. I need therapy for depression and anxiety. I think a lot of the way therapists are taught is that since transgender people have a higher rate of depression and anxiety and other mental health issues, people see it as the cause of those, and that's not the case. It's just like any other form of oppression, or bullying, or any of that. Not productive to your profession to frame it as the issue… Instead of making someone's gender identity the problem, you can make other people the problem… instead of making my gender identity the problem instead of how other people treat me. They make it so much about, ‘Oh, how do you identify? How is your transition going?’ Instead of like, ‘How are you feeling?’ It's not about transitioning, or being trans. It's about how I feel.
	Participant 9 spoke to inclusion on paperwork and forms, and the utility of accurate data in providing comprehensive medical care	I'd say starting something like they/them pronouns. Having a different option than just male or female, maybe just write in what your sex is. I don't know. Just having a certain qualifier where I can put down, “Yes, my birth sex is female. For the most part I'm female, but I'm also male, but I don't have any breasts, and I'm also taking testosterone.” Because it can be very confusing if somebody marks “man” on there, and they are a female bodied person. It can be confusing for medical reasons, so I think definitely having options for not only medical documents but for any type of document that would want that kind of demographic knowledge. I think it's pretty important.
Providers not informed: physically and emotionally taxing for participants Challenge systemic oppression (e.g., binary options only on forms, outdated language or practices, and need for facilities to be inclusive and welcoming) Societal expectations and provider bias: • toward transitioning (socially and medically) • assessment, presenting concerns, and treatment planning Providers need to listen, validate, and understand TGD people as both vulnerable and resilient. Transmasculine identity is not the core of distress, rather external stressors Transmasculine identity as a source of resilience (e.g., “gender euphoria”) Explore bias and consequences of binary and medicalization Positive patient–provider experiences can increase levels of safety, trust, validation, and access to competent care, thus reducing disparities	Participant 12 pointed to accessibility to trans affirmative care. In addition, his account demonstrated how misinformation is not limited to cisgender providers. Finally, Participant 12 takes a humanistic view on what it means to be a good therapist	Well, I was paying an arm and a leg before. Yeah, I was going to this place where it was $200 every few months for a blood test. Then I started going to this new place where they have trans doctors there, it's like a queer office, and they only charge you 60 bucks and it's freaking awesome. It's like a clinic. Like a trans clinic. It's really awesome. The best thing you can do is just listen, as far as therapists, and medical practitioners, too… because I've dealt with this. Not every transition is the same, and people cannot compare theirs. I had a doctor who was a trans woman, and she was terrible because she kept trying to compare our transitions and wanted me to do certain things because of it, and personally that's wrong. People are different and react to things differently. As far as therapists, they just need to listen. They need to understand that they don't know anything. The trans person that is in therapy knows more about their transitioning than that therapist does, most likely, because they live it they experience it. As much as a therapist wants to try, they will never know how it feels, so they just have to listen and accept what that person is saying.
	Participant 5 remarked on the power of language in maintaining or challenging oppression; expanding language will be liberating for all gender identities. In addition, expanding the narrative on TGD identities and increasing positive visibility will result in more accurate representations of TGD people's lives and resiliencies.	Yeah… we're limited by the language that we're using, you know, and we know that the language that we choose impacts how we think about things. It's not it's not like those two things can be separated from each other, so I think we do need to open up language significantly because everything is gendered in the binary right now, and it's not accurate or authentic … and it does harm to people who don't fall into it that. And it does harm to people who even do fall into that. But I'm really sick of every trans narrative being negative, and doom and gloom. It's like, here's a Buzz Feed article of trans people living in gutters. How about no. You know? Let's create a wider narrative of trans people. Like, I'm trans and I'm working in the field that I love and the way that I express my gender to the world makes me feel gender euphoria because I'm able to be authentic…being authentic makes you feel good about yourself. It makes you feel happy… the opposite of dysphoric. So I want us to latch onto those things.
	Participant 8 astutely and empathetically illustrated a learning process by which health care professionals can challenge essentialist views embedded in their socialization, widen the narrative, and deliver proficient services, research, and policies.	Well, it [trans masculine identity] challenges the idea of gender that people have been given since literally the day they were born. It kind of throws everything about gender out the window, and people don't like new things. They don't like their ideas challenged. It's like, in first grade when you learned about multiplication, and they had to explain it to you using addition because addition isn't scary. You've been learning about this for, like a year now, but then you go into multiplication and it's like, “Oh my God! What is this?” But really, it's not that bad. As soon as people start understanding it, it's not that bad.

**Table 6.** Recommendations for Health Care Clinicians, Researchers, Staff, and Systems

1. Active immersion with TGD individuals and communities
2. Educate providers, researchers, and systems on issues relevant to TGD health (e.g., workshops, trainings, didactics, and consultation)
3. Self-awareness and reflexivity of own biases in research, practice, and policy
4. Conduct affirmative research in multiple fields of TGD health (e.g., endocrinology, public health, mental health, neuropsychology, and primary care)
5. Include space for TGD demographics in research; understand sex and gender as related but separate constructs/variables—consider write-in options
6. Develop TGD-centric frameworks in policy, research, and practice—include TGD people in the process
7. Understand “transitioning” as a multifaceted and individualized lifelong process, which may or may not include medical intervention(s)
8. Update clinical forms and medical charting, reflecting a greater number of options for sex and gender identities
9. Ask about, and use, correct name and pronouns in all stages of a visit (e.g., front desk staff and providers)
10. Understand essentialist, binary underpinnings of diagnoses and treatment
11. Understand the pros and cons of past and current Standards of Care for TGD people
12. Understand the costs and benefits of gatekeeping and informed consent models of care
13. Understand TGD identities as a source of resilience vs. pathology—consider sources and effects of external stressors (e.g., transphobia and cissexism)
14. Do not assume TGD identities are the crux of all presenting concerns—listen and validate
15. Develop and advocate for affirmative resources and services for TGD people
16. Create TGD inclusive spaces (e.g., media and literature reflecting TGD identities, all gender restrooms, and value diversity in hiring practices)

Participants highly valued positive patient–provider interactions, which included the following: active listening, demonstrating competencies in TGD health, using correct name and pronouns, and welcoming spaces. Informed providers understood limitations and advocated for change in TGD health care. They were knowledgeable about informed consent versus gatekeeping models, and did not pathologize identity. They listened, rather than pushing a specific trajectory or treatment plan, and did not assume TGD identity as the core of presenting concerns. In fact, informed providers understood transmasculine identity as an individualized process and a source of immense resilience and strength.

## Discussion

Participants provided insights regarding transmasculine experiences in health care systems, revealed factors causing mistrust, and recommended measures to improve interactions. Subthemes included the following: (1) an essentialist, binary medical model is inaccurate and oppressive and (2) consequences of medicalizing gender (i.e., gender as a diagnosis). A third subtheme highlighted steps to improve TGD health care.

### Medical model in context

Understanding how sex and gender are governed through societal forces requires examining the historical relationship between medicine and TGD embodiment, including advancements in endocrinology, surgical interventions, and unethical experimentation. Extensive interdisciplinary review documents this history.^3,24–30^ Stryker explained how medicine's social authority and influence on TGD health have grown, potentially surpassing that of religion.^29^ Stryker^29^ acknowledged the positive impact of modern medicine in everyday life, but asserted medicine maintains and regulates normative standards, posing substantial obstacles for TGD individuals.

Likewise, Johnson explained, “Transgender identity and experience has been formally claimed and defined by medical authority since the introduction of a psychiatric diagnosis for gender variance in DSM-III.”^31^ Indeed, diagnostic classification in the DSM was revised from Gender Identity Disorder (DSM-III, 1980) to Gender Dysphoria (DSM-5, 2013).^32,33^ Revisions represented a shift in ideology focused on treating psychological distress rather than labeling TGD identity a disorder. However, the individual and their embodiment remain the crux of distress.^31^ Notably, our data indicated that gender-related psychological distress stemmed predominantly from external sources of transphobia and cissexism. Finally, the current diagnosis acknowledges not all transgender individuals experience dysphoria, but presumes medical intervention is the next step for those who do,^31^ and diagnostic language reifies an essentialist binary (e.g., diagnostic criterion: “A strong desire to be of the other gender”).^33^

Overall, standards of care have progressed, as evidenced by those set forth by WPATH.^15^ However, gatekeeping persists. For example, as noted,^31^ WPATH^15^ included a revision stipulating that individuals 18 years of age and older should be granted access to care based on informed consent. However, providers are not mandated to uphold these practices, and maintain great power in governing TGD identities. In addition, despite revisions to the DSM-5^33^ and ICD-11,^16,17^ reframing systemic policies, cultural norms, and health professionals' perspectives will be a process that requires insight and time.

### Tangible inlets for reducing health disparities

Participants' negative experiences could be understood through the Minority Stress Model, which Meyer^9^ characterized as (1) unique and additive, (2) chronic, and (3) socially devised/maintained. Marginalized people must continually adjust to additive and pervasive stressors not experienced by nonstigmatized individuals. Embedded in a sociocultural context, the stress is chronic and unrelenting, and may lead to negative biopsychosocial health outcomes. Finally, marginalization and resultant stress are perpetuated through socially derived institutions and systems (e.g., health care) outside of one's control, devised to maintain existing power hierarchies.

In addition, the Weathering Hypothesis^11^ proposes that accumulated burden from confronting multiple, on-going psychosocial stressors manifests as accelerated aging. The related theories of minority stress and weathering have been independently tied to adverse health effects (e.g., allostatic load, diabetes, cardiovascular disease, depression, and post-traumatic stress disorder) in people with marginalized identities.^34–43^ Researchers are beginning to include TGD individuals in studies exploring the mental and physical health effects of minority stress and weathering.^5,34,35,44^

Our data illustrate how minority stressors and weathering ensue through patient–provider interactions, offering tangible inlets to reduce health disparities. For example, consistent with NTDS^5^ findings, our participants described having to repeatedly educate providers steeped in ciscentric, binary views. They described this process as physically and emotionally taxing, exemplifying minority stress^9^ and potentially invoking the weathering process.^11^ Despite pleas for competent health care, essentialism in the medical system reflects an inherent bias toward caring for cisgender individuals, prompting TGD individuals to avoid health care rather than risk invalidating oppressive and unsafe interactions.

In addition, marginalization and subsequent minority stressors and weathering processes are perpetuated through medicalizing gender and gatekeeping practices (i.e., requirement of a pathological, stigmatizing diagnosis and multiple letters of support to access care).^34,45^ Ciscentric and essentialist reference points in the medical approach link interventions to diseases and disorders,^24,28,31,46^ perpetuate social expectations that cisgender identity is the normal and valid standard, and serve as bedrock for participants' fears around being perceived “not trans enough.” For example, Benjamin^47^ was among the first physicians to offer hormonal and surgical options for trans-identified people. However, consistent with the medical zeitgeist, he labeled “true” trans people as those who desire medical intervention to “align” their physical bodies with their gender identity.^48^

This message persists today, as our participants experienced repeated stressors and unrelenting pressure to align with cisgender conceptions of male/masculine to appease gatekeepers.^45,49^ For our transmasculine participants, “not trans enough” centered around expectations to conform to socially constructed standards of masculinity to be deemed valid. Moreover, participants reported a double standard in following prescribed cisgender trajectories. For example, feminine cisgender men may be targets for discrimination; however, their gender identity and sex are not questioned as is common with transmasculine people who demonstrate similar qualities.^50^

Entwined with the Benjamin standards,^47^ “not trans enough” is steeped in essentialism and perpetuated through authoritative frameworks, such as multiple iterations of the Diagnostic and Statistical Manual of Mental Disorders (DSM).^32,33,51^ Davis *et al*^.8^ characterized the Institute of Medicine's influence in creating and disseminating ideologies around sex, gender, and sexuality as such, “… they not only perpetuate but produce the notion that a healthy body is identifiably male or female, masculine or feminine, and heterosexual.”

This creates tension, as providers have the power to bestow sex and gender, as well as reify the status quo. In sum, the consequences of essentialism in medicine are fundamental to participants' negative experiences, resulting in minority stress, inaccurate diagnostic assessment and treatment, increased vulnerability, distrust, and avoidance of health care. Thus, it is imperative that practitioners and researchers examine socialized biases. In doing so, we have power to minimize deleterious effects of compounding distal minority stressors and weathering processes on TGD people, and maximize outcomes in TGD health.

More than half of participants described burdensome nuances of minority stress associated with viewing TGD identities as a pathology, and subsequent gatekeeping practices. To elaborate, letters from physicians and psychologists are often required to begin legal processes of amending identity documents (e.g., driver's license, and passport). Many U.S. states require a surgeon's letter to amend assigned sex on birth certificates^45,49^ In other words, some form of binary-based surgical intervention must be performed, regardless of individual preferences or socioeconomic access. Interestingly, the process of gatekeeping may inflate or misrepresent rates of TGD individuals seeking psychotherapy, their presenting concerns, and rates of gender dysphoria diagnoses.^49^

In addition, some participants who desired medical intervention(s) sidestepped gatekeeping (i.e., adapting to minority stress) by finding clinics offering services based on informed consent. Participants understood this access as a privilege, closely tied to other facets of identity (e.g., socioeconomic status, race, and geographic location). While participants were not specifically asked about their level of access to health insurance coverage, disclosures were intertwined in many participant responses. Overall, participants demonstrated a high level of insight and awareness regarding privileges they benefitted from, including access to health insurance and/or any degree of gender-related service coverage. However, despite most participants having access to health care, most expressed added burdens, such as facing discrimination, while having to navigate a complicated system on their own, or repeatedly educating providers around gender identity and sex. For this reason, trust may be difficult to establish in a variety of settings, including educational settings that frequently rotate providers (e.g., hospital residents and interns).

Practitioners' assumptions and pathology-based framework contribute to an essentialist, gatekeeping model that imposes minority stressors detrimental to TGD health.^28,52^ However, consistent with the literature,^24,28,31,46,53^ our participants challenged pathological notions of TGD identity by reporting ways transmasculine identity serves as a source of pride, well-being, and resilience that can buffer the negative effects of minority stress and weathering.^35^ Moreover, participants rejected the idea that one must be dysphoric to benefit from medical intervention. As one participant explained, one who is “euphoric” may also desire medical options. Participants challenged the “one and done” conceptualization of transitioning, explaining that transition is a multifaceted psychosocial developmental process that may or may not include any number of medical interventions.

In response, TGD health experts consistently advocate for nonpathological frameworks to conceptualize gender identity development, evidence-based care, and access to affirmative care, including informed consent.^15,28,52^ Revisions to the ICD-11^16,17^ will inevitably bring changes on an international scope, but the directions of change have yet to be seen, and shifting the landscape to a non-pathological framework will take time.

In the meantime, providers can take practical steps to reduce the significant and cumulative burden of minority stress, as reported by participants in this study (Tables 5 and 6). Providers can acknowledge and challenge socialized biases, educate themselves, immerse themselves, and work with TGD communities to advocate for change in research, practice, and policy. Providers can be transparent with patients, acknowledging strengths and limitations of current systems. Providers can be knowledgeable in TGD health research, pass this information along to patients, and collaboratively support them as they make informed decisions around their health care.

### Strengths and limitations

Strengths of IPA methodology included the ability to develop research questions relevant to transmasculine health, and recruit participants accordingly. The idiographic focus enabled detailed themes and patterns to emerge across participants.^22^ However, given this research was based on the experiences of 12 self-identified transmasculine young adults 18–35 years of age from urban and rural areas in the Midwest, most with access to health care and some degree of college education, caution must be used when generalizing insights. Twelve participants generated an extraordinarily large data set for IPA, and the richness of idiographic analysis may have been compromised in favor of thematic group interconnectedness. However, the benefits of IPA outweighed the limitations, and future research will enable wider generalizations over time.^22^

## Conclusions

This research illustrated and honored the lived experiences of transmasculine individuals. In this study, we focused on one superordinate theme, Perspectives on Health Care, with three subthemes: (1) An essentialist, binary medical model is inaccurate and oppressive,(2) consequences of medicalizing gender (i.e., gender as a diagnosis), and(3) recommendations to improve health care.

To access health care, transmasculine individuals need not invoke a narrative that is linear, binary, and reflective of cisgender standards. In addition, practitioners wield great power to address miseducation, such as transition being synonymous with a one-and-done medical cure for a pathological condition. Moreover, validating TGD identities decreases stigma, inevitably influencing positive mental and physical health outcomes. Participants' gender identity can be a substantial source of strength, just as it can for anyone. Thus, it is imperative practitioners examine their own power and biases, and broaden conceptualizations in research and clinical practice. Neglecting to do so denies TGD people the dignity and care they deserve, and perpetuates health disparities. Active engagement will positively impact people of all genders, and help build frameworks that promote the well-being of TGD people. This is within our ethical code in research and practice, as we must first do no harm.

## Abbreviations Used

IPA: Interpretive Phenomenological Analysis
NTDS: National Transgender Discrimination Survey
TGD: Transgender and Gender Diverse
WHO: World Health Organization
WPATH: World Professional Association for Transgender Health
